# The Positive Rate of Nucleic Acid Testing and the Epidemiological Characteristics of COVID-19 in Chongqing

**DOI:** 10.3389/fmed.2021.802708

**Published:** 2022-01-14

**Authors:** Xiaohua Liang, Yajun Sun, Lun Xiao, YanLing Ren, Xian Tang

**Affiliations:** ^1^Department of Clinical Epidemiology and Biostatistics, Children's Hospital of Chongqing Medical University, National Clinical Research Center for Child Health and Disorders, Ministry of Education Key Laboratory of Child Development and Disorders, Chongqing Key Laboratory of Child Health and Nutrition, Chongqing, China; ^2^Center for Disease Control and Prevention of Jiulongpo District, Chongqing, China

**Keywords:** COVID-19, close contacts, suspected cases, asymptomatic carrier, positive rate

## Abstract

**Objective:**

The purpose of this study is to analyze the positive rate of severe acute respiratory syndrome coronavirus 2 (SARS-CoV-2) nucleic acid testing (NAT), cases of and deaths due to SARS-CoV-2, and the epidemiological characteristics of SARS-CoV-2 to identify high-risk populations.

**Methods:**

A retrospective study in Jiulongpo district of Chongqing was conducted by performing continuous observations of the frequency of SARS-CoV-2 NAT, analyzing the data of close contacts of patients and asymptomatic carriers, and collecting epidemiological data. Data were collected from January 20, 2020, when the first case of SARS-CoV-2 infection was reported, to March 26, 2020. Descriptive statistical analysis and Cochrane–Mantel–Haenszel analysis were used to compare the positive detection rates and positive diagnostic rates of different exposure groups.

**Results:**

A total of 7,118 people received 10,377 SARS-CoV-2 nucleic acid tests in one district, and the SARS-CoV-2 positive rates were 0.40% (18/4446) and 0.15% (4/2672) in people receiving one and ≥ two nucleic acid tests (*p* = 0.06), respectively. Those with suspected cases (12.35%) and close contacts (8%) had higher positive rates than people tested at fever clinics (0.39%) (*p* < 0.001). The median latency (range) of cases was 5 (2, 9) days, and the median time from diagnosis to recovery was 22 (14, 25) days. One recovered patient received a positive test result at 28 days after recovery when she attempted to donate blood. Six clustered cases, including one patient who died, indicated persistent human-to-human transmission. One patient who was diagnosed after death was found to have infected 13 close contacts. People working in catering and other public service departments (36.36%) and people who are unemployed and retirees (45.45%) have an increased risk of infection compared with technical staff (9.09%) and farmers (9.09%).

**Conclusion:**

The total positive rate was low in the tested population, and more effective detection ranges should be defined to improve precise and differentiated epidemic control strategies. Moreover, in asymptomatic carriers, SARS-CoV-2 tests were positive after recovery, and patients with suspected SARS-CoV-2 infection who die may pose serious potential transmission threats.

## Key Notes

The total positive rate was low in the tested population, whereas the positive rates of suspected cases and close contacts were high. In asymptomatic carriers, SARS-CoV-2 tests were positive after recovery, and patients with suspected SARS-CoV-2 infection who die may pose serious challenges to the prevention and control of SARS-CoV-2.

## Introduction

In December 2019, severe acute respiratory syndrome coronavirus 2 (SARS-CoV-2) emerged in Wuhan, China. With the spread of SARS-CoV-2, causing the coronavirus disease 2019 (COVID-19) epidemic, cases were identified in other locations in China and many other countries worldwide. COVID-19 was declared a public health emergency of international concern by the World Health Organization, and it progressed to a pandemic associated with substantial morbidity and mortality (1). COVID-19 is highly contagious and transmitted mainly through the respiratory tract and close contact with infected individuals (2, 3). As SARS-CoV-2 is a new infectious agent, it spreads rapidly, and the rapid development of scientific prevention and control strategies has been challenging. As of March 26, 2020, there were 82,034 confirmed cases and a total of 3,293 deaths nationwide in China; 50,006 cases were from Wuhan, according to the official website of the National Health Commission of the People's Republic of China (http://www.nhc.gov.cn/). Chongqing, which has a population of 38 million people, borders Wuhan; however, initial cases were mainly imported from Wuhan. As of March 26, 2020, there were 578 confirmed cases (including two imported cases from abroad), with 6 (1.04%) deaths. At the end of February, the SARS-CoV-2 epidemic was successfully contained through public health interventions such as case detection, isolation, and movement restrictions (4); new cases have not been reported since February 26, 2020, according to the statistics of the Chongqing Municipal Health Commission (http://wsjkw.cq.gov.cn/). China's health service system has played an important role in epidemic prevention and control. In China, the health care system is comprised of two sections, medical institutions (hospitals, primary medical and health centers, such as township hospital, or community health center) and public health organizations, such as Centers for Disease Control (CDCs) and Centers of Health Supervision; these medical organizations are stratified into five levels: state, province, city, county/district, and town (5). After the start of the pandemic, the Chinese government released pandemic control policies under a “unanimous nationwide system” to joint defense and control by multiple departments (6).

Many studies on COVID-19 have focused on the pathogen (7), transmission routes, clinical diagnosis, and treatment methods (8). Various public health measures have been successfully implemented at different stages of the SARS-CoV-2 epidemic worldwide (9–12). China successfully contained the outbreak through strict lockdown measures (13, 14). High-income countries, such as New Zealand and Australia, eliminated community transmission for several months during 2020 through strict border control and extensive contact tracing (15). Despite recent advances in vaccine development, nonpharmaceutical interventions will remain paramount until the very end of the pandemic (9). A previous study comprehensively evaluated the association of various public health interventions implemented by the Chinese government (including but not limited to intensive intracity and intercity traffic restrictions, social distancing measures, home isolation and centralized quarantine, and medical resource improvements) with outbreak control within Wuhan city (13) and found that in the absence of either effective drugs or a vaccine, robust, multifaceted containment, mitigation, and suppression measures were temporally associated with improved epidemic control. A study that examined changes in infection rates in 15 states and the District of Columbia before and after mask mandates showed that rates were increasing before the mandates were implemented and slowed significantly after, with greater benefits over time (16); several other studies reported the same conclusion (17, 18). Recent studies have found that multilayer cloth mask use, increased social distancing, and eye protection use are associated with lower rates of infection (17, 19). In addition, nucleic acid testing (NAT) is an effective tool to identify positive cases and provide clues to the source of infection. At present, there are two diagnostic methods for SARS-CoV-2 detection: serological testing and NAT (20, 21). Serologic tests, which directly detect antibodies or antigenic viral proteins, can yield rapid results (22, 23), but they only accurately detect one-half to three-quarters of infections, with the possibility of false-negative results, especially among asymptomatic individuals (24). NAT includes quantitative reverse transcription polymerase chain reaction (qRT-PCR) (25), isothermal amplification, and clustered regularly interspaced short palindromic repeats (CRISPR) technology (26, 27) and is more accurate than serological testing. To enhance the efficiency of NAT in large-scale screening, researchers pool (or combine) samples for testing (28, 29). Sample pooling, a strategy used for early and comprehensive screening for influenza virus and human immunodeficiency virus (HIV) (30), and now SARS-CoV-2 (31–33), has been shown to be a cost-effective method for large-scale diagnostic testing and also community screening with good test accuracy; the assay relies on a Y-double probe modified on g-FET, targeting both the open reading frame 1ab (ORF1ab) and N genes of SARS-CoV-2 nucleic acids, enabling high recognition rates and detection limits (0.03 copy muL-1) that are 1–2 orders of magnitude lower than those of existing nucleic acid detection methods. This method achieves the fastest nucleic acid detection (1 min) and has allowed the first direct 5-in-1 pooled assay (34, 35). One study identified the group size of the pooled assay and subsequently compared the pooled assay with individual assays; the study established that the sensitivity of the pooled assay was similar to that of individual assays (36).

It is critical to identify the most effective public health interventions for different phases of the epidemic. In the beginning, expanding the scope of quarantine inspections increased the burdens of local Centers for Disease Prevention and Control (CDCs) and other medical institutions and reduced positive detection rates. In addition, many studies focused on the effects of various public health intervention strategies (i.e., public activity bans and internal movement restrictions), but limited studies explore the positive test rates of SARS-CoV-2 screening in different risk exposure populations. Moreover, no study has analyzed how to carry out epidemiological investigations of fatal cases. For the early stage of infectious disease outbreaks, effective prevention and control standards have not been established, so it is of great significance to allocate medical resources reasonably, as allocating medical resources with high efficiency can effect the prevention and control outcomes. In addition, it is important to focus on populations with the highest risk and ensure an appropriate epidemiological investigation scope for regular epidemic prevention and control in China, especially when addressing the challenges of SARS-CoV-2 variants in 2021. Accordingly, our study adds new evidence on how to effectively control the spread of emergent infectious diseases, including NAT and detecting the chain of transmission by decedents.

## Methods

### Sampling the Target County

Three criteria were considered in selecting the survey district for this study. First, there were enough residents and cases in the target district; 1.2 million people permanently resided in the surveyed district and over 20 cases of COVID-19 were diagnosed. Second, the chosen district contained both urban and rural areas to eliminate the effects of urban–rural differences. Last, the hospitals in the district could afford the total medication demands for patients with COVID-19 and residents in isolation. Therefore, in Chongqing, the target county met al.l the three criteria, with good representation.

### Epidemiological Data Collection

Epidemiological data on COVID-19 in Chongqing were collected from the official website of the Chongqing Municipal Health Commission (http://wsjkw.cq.gov.cn/) from January 20, 2020, when the first case of COVID-19 was reported, to March 26, 2020. The data included daily new cases, suspected cases, cumulative cases, hospitalized cases, severe cases, deaths, and discharged cases. Epidemiological follow-up survey and diagnostic detection test data were obtained from the CDC of Jiulongpo district in Chongqing. Policy materials on public health interventions for COVID-19 were collected from the official websites of both the Chongqing Municipal Health Commission and the Chongqing Municipal Government. The Institutional Review Board at the Children's Hospital of Chongqing Medical University provided approval for this study.

### Diagnostic Criteria

The Protocol on Prevention and Control of Novel Coronavirus Pneumonia (Edition 6) (37) was used for the diagnosis of cases. The diagnostic criteria for suspected cases, confirmed cases, asymptomatic infections, case clusters, and close contacts were as follows (37). For suspected cases, at least one of the following epidemiological histories was required: (1) a history of travel to or residence in Wuhan or its surrounding areas, other communities in China where cases had been reported, or other countries or areas with severe outbreaks, within 14 days prior to the onset of the disease; (2) contact with a person infected with SARS-CoV-2 (with a positive NAT result) within 14 days prior to the onset of the disease; (3) contact with patients with fever or respiratory symptoms from Wuhan or its surrounding area, communities where confirmed cases had been reported, or other countries or areas with severe outbreaks within 14 days before the onset of the disease; or 4) part of a cluster (2 or more cases with fever and/or respiratory symptoms in a small population, such as family, office colleagues, classmates, workshop attendees, etc., within 14 days). Additionally, at least two of the following clinical manifestations were required: (1) fever and/or respiratory symptoms; (2) imaging characteristics of novel coronavirus pneumonia; or (3) a normal or decreased white blood cell (WBC) count and/or a normal or decreased lymphocyte count in the early stage of onset. A suspected case was defined as any one of the epidemiological history criteria plus any two clinical manifestations or all three clinical manifestations if there was no clear epidemiological history. Confirmed cases were defined as suspected cases with one of the following etiological or serological results: (1) real-time fluorescent RT-PCR positivity for SARS-CoV-2 nucleic acid; (2) detection of a viral gene sequence highly homologous to the known SARS-CoV-2 sequence; (3) SARS-CoV-2-specific Ig M and IgG detected in serum; or a SARS-CoV-2-specific IgG titer was at least 4-fold higher during convalescence than during the acute phase. Asymptomatic infections were defined as SARS-CoV-2 virus detected in respiratory specimens or IgM detected in serum. Asymptomatic cases were mainly found through close contact tracing, investigations of clusters, and infection source tracing. Case clusters were defined as two or more confirmed cases or asymptomatic patients within a small area, such as family homes, offices, schools, workshops, etc., within 14 days, with the possibility of human-to-human transmission or a common exposure source. Close contacts were defined as those who had unprotected close contact with a patient with a confirmed or suspected case within 2 days before illness onset or with an asymptomatic infected person within 2 days before sampling.

The presence of SARS-CoV-2 in respiratory specimens was detected by real-time RT-PCR amplification of SARS-CoV-2 ORF1ab and nucleocapsid protein (NP) gene fragments using kits provided by Beijing Zhuochenghuisheng Biotechnology Co., Ltd. and Da An Gene Co., Ltd. The conditions for amplification were 50°C for 15 min, 95°C for 3 min, and 45 cycles of 95°C for 15 s, and 60°C for 30 s. When the two targets (ORF1ab and NP) were amplified by specific real-time RT-PCR from the sample, the case was considered to be laboratory-confirmed.

### Statistical Methods

Data were double entered into Microsoft Access by two people using a blinding method, and a consistency check was performed before analysis. Differences in anthropometric variables with a normal distribution between the two groups were assessed using Student's *t*-test. Continuous variables that did not have a normal distribution were expressed as X50% (X25%, X75%), and the Wilcoxon rank sum test was used for comparison between the two groups. All continuous variables are expressed as the mean ± standard deviation (SD) if they satisfied a normal distribution. The positive rates of SARS-CoV-2 are reported as numbers (n) and percentages of the total (%) and their 95% confidence intervals (CIs). The chi-squared test was used to detect differences, and the Bonferroni method was used for *post hoc* analyses among the three groups. Cochrane–Mantel–Haenszel analysis was used to compare the positive detection rates and the positive diagnostic rates of different exposure groups. A significant difference was determined at an α-level of 0.05. Data analysis in this study was conducted using SAS 9.4 software (Copyright©2016 SAS Institute Inc. Cary, NC, USA). A significant difference was defined by a two-sided α-level of 0.05.

## Results

### SARS-CoV-2 Nucleic Acid Detection and Positive Rate

The surveyed county is located in southwestern Chongqing. It covers an area of 432 square km and contains eight streets and 11 towns. It has a permanent population of ~1.2 million people. As of March 27, 4,952 (0.41%) local people and 2,166 travelers had received SARS-CoV-2 NAT. In total, 7,118 people received 10, 377 SARS-CoV-2 tests; of them, 4,446 (62.46%), 2,334 (32.79%), 154 (2.16%), 133 (1.87%), and 51 (0.72%) people were tested one, two, three, four, and more than four times, respectively (Table 1). Five people received more than nine SARS-CoV-2 tests, and one person received 12 SARS-CoV-2 tests. Moreover, SARS-CoV-2 NAT was positive after death in one person.

**Table 1:** Time of SARS-CoV-2 NAT in Jiulongpo district of Chongqing.

**Variable**	**Total samples**	**Detected samples**	**Time of detection**
Total	7,118	7,118	10,377
*Detection numbers*			
First detection	7,118	4,446 (62.46%)	4,446 (42.84%)
Second detection	2,672	2,334 (32.79%)	4,668 (44.98%)
Third detection	338	154 (2.16%)	462 (4.45%)
Fourth detection	184	133 (1.87%)	532 (5.13%)
More than four times		**51 (0.72%)**	**292 (2.81%)**
Fifth detection	51	37 (0.52%)	185 (1.78%)
Sixth detection	14	8 (0.11%)	48 (0.46%)
Seventh detection	6	1 (0.014%)	7 (0.067%)
Eighth detection	5	0 (0%)	0 (0%)
Ninth detection	5	1 (0.014%)	9 (0.087%)
Tenth detection	4	2 (0.028%)	20 (0.19%)
Eleventh detection	2	1 (0.014%)	11 (0.11%)
Twelfth detection	1	1 (0.014%)	12 (0.12%)
*Population sources*			
Fever clinic	—	2,803 (39.38%)	—
Close contacts	—	397 (5.58%)	—
Suspected cases^#^	—	144 (2.02%)	—
From Hubei^*^^#^	—	1,971 (27.69%)	—
From other provinces	—	7 (0.10%)	—
From abroad^#^	—	184 (2.58%)	—
Correctional officers and prisoner	—	1,249 (17.55%)	—
People in pension agency	—	82 (1.15%)	—
Other sources	—	281 (3.95%)	—

* *From Hubei after February 20, Wuhan is the capital city of Hubei Province*.

# *Diagnosed cases were not included if they were included in other categories*.

*The bold values represent the total samples*.

The positive rates of COVID-19 are shown in Table 2. The positive rate of COVID-19 was 0.31% (0.18%, 0.44%) in the total tested samples. The positive rates were different for different high-risk groups. The positive rates were 0.39% (11/2803), 8% (30/397), and 12.35% (21/170) in people who attended fever clinics, close contacts, and people with suspected cases, respectively (*p* < 0.05). The positive rates were 0.40% (18/4446) and 0.15% (4/2672) in people who received one and ≥ two nucleic acid tests, respectively (*p* = 0.06). In addition, the positive rates were 0.34% (17/4952) and 0.23% (5/2166) in local residents and travelers, respectively (*p* = 0.509). Moreover, the positive rates of COVID-19 were 0.20% (4/1974), 12.25% (1/8) and 0 for those with exposure to Hubei Province (*p* = 0.024), other provinces and abroad, respectively. Among the locally detected samples, the positive detection rates were 0.51% (17/3340) and 0 in samples from special institutes and other local samples, and the difference was significant (*p* < 0.01).

**Table 2:** The positive detection rate of COVID-2019 from different sources.

**Detection sources**	**Total**	**Negative cases**	**Positive cases**	**Positive rate (95% CI)**	***P***
**High-risk population**					
Fever clinics	2,803	2,792	11	0.39% (0.16%, 0.62%)	<0.001
Close contacts	397	367	30^*^	8.00% (4.96%, 10.16%)^a^	
Suspected cases	170	149	21	12.35% (7.41%, 17.30%)^a^	
**Local close contacts**	360	330	30	8.33% (5.48%, 11.19%)	
Living in Jiulongpo district	164	150	14	8.54% (4.26%, 12.81%)	0.898
Living in other places	196	180	16	8.16% (4.33%, 12.00%)	
**Number of tests before diagnosis**					
One time	4,446	4,428	18	0.40% (0.22%, 0.59%)	0.062
≥Two times	2,672	2,656	4	0.15% (0.00%, 0.30%)	
**Different sources**					
Local samples	4,952	4,935	17	0.34% (0.18%, 0.51%)	0.509
Imported samples	2,166	2,161	5	0.23% (0.03%, 0.43%)	
**Imported samples**					
From Hubei province	1,974	1,970	4	0.20% (0.00%, 0.4%)	0.024
From other places in mainland China	8	7	1	12.5% (0.00%, 35.42%)	
From abroad	184	184	0	0%	
**Local samples**					
Other local samples	3,340	3,323	17	0.51% (0.27%, 0.75%)	0.004
Special institutes in local area^#^	1,612	1,612	0	0%	

* *Included cases in other counties*.

# *Correctional officers and prisoners, people in pension agencies and other institutions; NAT, nucleic acid testing*.

a *Represent a significant difference compared with cases from “Fever clinics"*.

### Characteristics of 21 Local Cases and One Asymptomatic Case

**Figure 1:**
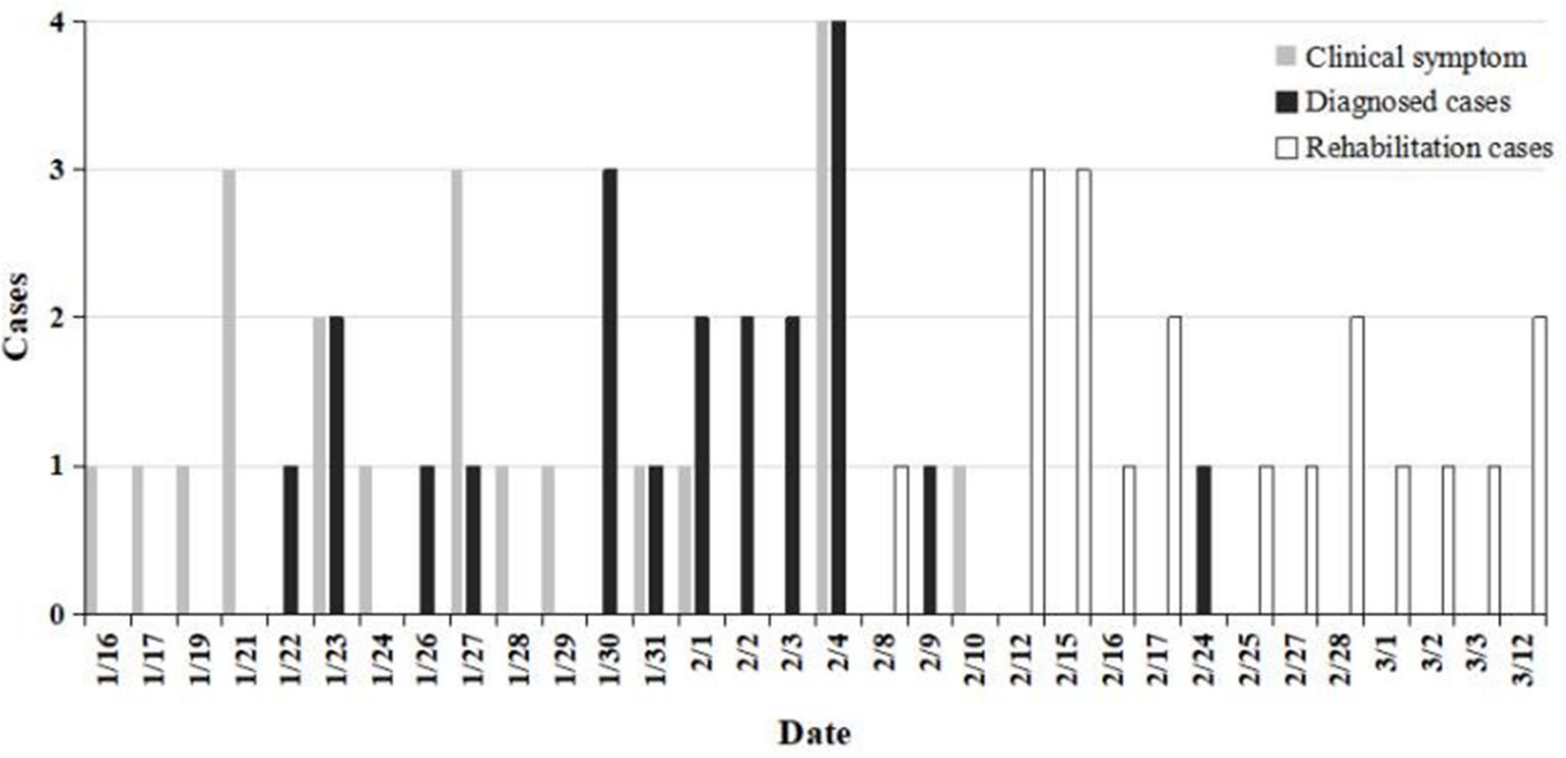
Diagnosis and recovery periods of 21 symptomatic patients.

Twenty-one patients with COVID-19 and one patient with asymptomatic COVID-19, with a median age of 51 (43, 57) years, were diagnosed in one district of Chongqing; 36.36% (8/22) of the patients were men (Table 3). Twenty patients experienced clinical symptoms between January 16 and February 4 and were diagnosed between January 22 and February 9 (Figure 1). Eighteen patients were diagnosed with one nucleic acid test, and four patients were diagnosed after two or three tests. Four and 12 patients were tested two and three times during the treatment period, respectively.

**Table 3:** The characteristics of 22 local cases in Jiulongpo district.

**Variables**	**N**	**Percentage**
**Sex**		
Male	8	36.36%
Female	14	63.64%
**Number of tests before diagnosis**		
One	18	81.82%
Two or three	4	18.18%
**Total number of tests per person**		
Two	4	18.18%
Three	12	54.55%
Four	3	13.64%
Five or six	3	13.64%
**Clustered cases**		
No	1	4.55%
Yes	**21**	**95.45%**
Cluster with one	2	9.09%
Cluster with two	4	18.18%
Cluster with three	2	9.09%
Cluster with four^*^	11	50.0%
Cluster with five^*^	1	4.55%
Cluster with six^*^	1	4.55%
**Case sources**		
Local cases	17	77.27%
Wuhan	4	18.18%
Other places	1	4.55%
**Diagnosis sources**		
CDC epidemiological survey	10	45.45%
Fever clinic	12	54.55%
**Occupation**		
Catering or public services	8	36.36%
Technical staff	2	9.09%
Unemployed, retiree	10	45.45%
Farmer	2	9.09%
**Rehabilitation status** ^**#**^		
Recovered	**20**	**95.24%**
Recovery from mild and common cases	19	95%
Recovery from severe cases	1	5%
Death	**1**	**4.76%**

* *Transferred 2, 1, and 1 cases from other districts, respectively*.

# *Excluded one asymptomatic carrier*.

*The bold values represent the total samples*.

**Figure 2:**
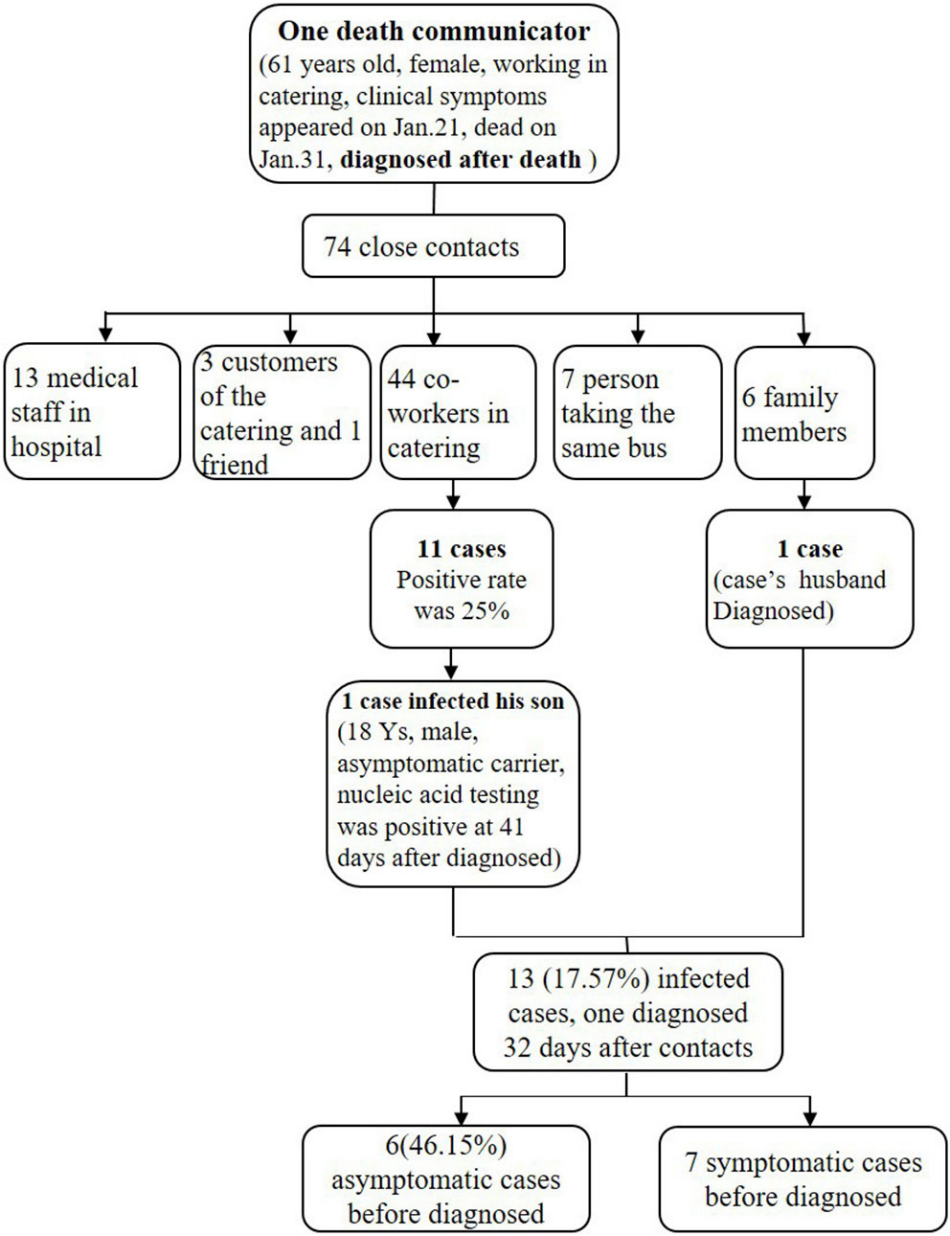
Transmission by one decedent.

Six cluster outbreaks were confirmed. One patient who was diagnosed after death infected 12 close contacts (Figure 2). The CDC surveyed 74 close contacts of the decedent, including 13 medical staff, three customers, one friend, seven people who rode the same bus, six family members, and 44 coworkers. One family member (husband of the decedent) and 11 coworkers were infected, and one coworker infected his son (asymptomatic carrier). The infection rates for all 74 close contacts and the 44 coworkers of the decedent were 17.57% (13/74) and 25% (11/44), respectively, which were both higher than the infection rate of all close contacts (8.00%, 30/397) (*p* = 0.01 and *p* < 0.001). Six (46.15%) infected patients were asymptomatic before diagnosis, and one close contact was diagnosed 32 days after contact.

There were four patients from Wuhan, one patient from another province and 17 patients from Jiulongpo district. According to occupation, eight patients worked in catering or public services, two patients were technical staff, 10 patients were unemployed or retirees, and two patients were farmers. The recovery rate was 95.24% (1/21); one case was severe, and one patient died before diagnosis. The incubation period was five (interquartile range: 2, 9) days, and the duration from diagnosis to recovery was 22 (interquartile range 14, 25) days. A nucleic acid test was positive after 28 days (including 14 days of centralized isolation) in one patient (24 years old) who had recovered, and SARS-CoV-2 positivity was found when she donated blood. An anal swab was positive in an asymptomatic carrier (18 years old) at 41 days after diagnosis.

### Epidemiological Survey and Close Contact Management

#### Close Contact and Suspected Case Management

Close contact management was reinforced during the SARS-CoV-2 pandemic. Before publication of the Protocol on Prevention and Control of Novel Coronavirus Pneumonia (Edition 6) (37), close contacts were defined as those who had close contact with patients with confirmed cases; after February 7, in the 6th edition, they were defined as those who had close contact with people with both suspected and confirmed cases.

A total of 360 close contacts were generated by the epidemiological investigation of diagnosed cases in Jiulongpo district (in Table 2). A total of 164 (45.56%) close contacts lived in Jiulongpo district, and 196 (54.44%) close contacts lived in other districts. A total of 8.33% (30/360) of close contacts were confirmed to be infected with SARS-CoV-2; 8.54% (14/164) of close contacts living in Jiulongpo district were diagnosed with SARS-CoV-2 infection, 8.16% (16/196) of close contacts living in other districts were diagnosed with SARS-CoV-2 infection, and one asymptomatic person (0.61%, 1/164) was positive for SARS-CoV-2 infection. The positive detection rate among suspected cases in the local county was 12.35% (21/170) (one positive carrier and all confirmed patients had suspected cases before diagnosis).

#### Public Security Monitoring of Close Contacts

Epidemiological investigation and public security technical analysis were used to monitor the patient's activity trajectory; the time was accurate to the exact minute, and the public transportation routes of people with suspected cases were announced after concealing personal information. To identify the close contacts of those with confirmed cases among the public, 5,450 bits of information of people who traveled on the same vehicle as those with confirmed cases were provided *via* the public security data. The CDC conducted a one-on-one survey of high-risk persons and identified close contacts. Centralized medical management was implemented for the high-risk close contacts, and general contacts were notified about their risk of SARS-CoV-2 infection through a service that delivered short messages.

Among the 638 registered close contacts (389 people with contact with local persons with confirmed or suspected cases, and 249 people with contact with people with confirmed or suspected cases from other districts) living in Jiulongpo district, 66 people were identified by public security data, and 360 and 212 people were found through epidemiological surveys performed by the Jiulongpo CDC and CDCs in other districts (Table 4). The discovery percentage for public security data was 10.3% (66/638); these data are an important supplement to CDC data.

**Table 4:** The relationships of close contacts in Jiulongpo district.

**Variables**	Total close contacts^*^	**Close contacts of local patients**	**Close contacts of patients from other places**	***p***
Total	638	360	278	
Relatives or friends	346 (54.23%)	236 (65.56%)	110 (39.57%)	<0.001
Strangers with contact by accident	277 (43.42%)	124 (34.44%)	153 (55.04%)	
Unconfirmed relationship	15 (2.35%)	0 (0%)	15 (5.40%)	

* *Close contacts of local patients or local patients with suspected cases and patients from other locations*.

The relationships between the close contacts and the patients were analyzed. Among the 638 close contacts registered in Jiulongpo district (including 389 close contacts of patients in Jiulongpo district and 249 close contacts of patients from other locations), 346 (54.23%) were relatives or friends, and 277 (43.42%) were strangers (accidental contacts). The relationships of 15 (2.35%) people could not be confirmed. In addition, among the 360 close contacts identified by epidemiological surveys, 236 (65.56%) were relatives or friends, and 124 (34.44%) were accidental contacts.

## Discussion

Effective public health interventions, such as the “Wuhan lockdown,” case detection, isolation, and movement restrictions, helped to control the SARS-CoV-2 pandemic. The positive rate of SARS-CoV-2 NAT (0.30%) in Jiulongpo district was low; the positive rate for ≥ two tests was lower than that for one test, and the positive rates of samples from close contacts and those with suspected cases were higher than that in samples from fever clinics. People working in catering or public services or people who are unemployed or retired have an increased risk of infection. The median incubation period was five (interquartile range: 2, 9) days, and the median time from diagnosis to recovery was 22 (interquartile range: 14, 25) days. Clustered cases indicated human-to-human transmission. Some patients who recovered became positive again according to NAT; these patients had asymptomatic cases and were considered asymptomatic carriers in the community. Moreover, one person with a suspected case died. These situations are key in the further control of the SARS-CoV-2 epidemic.

This study found that the positive rates of NAT were different among different high-risk populations. The positive rate was low for the total tested samples in Chongqing, whereas the positive rate was 12.35% in people with suspected cases in Chongqing. However, one study reported that the positive rate was 38% among a total of 4,880 specimens; 57% of patients visiting the fever clinic in a hospital in Wuhan were positive, and male and older populations had higher positive rates than female and younger populations, respectively (38). The different positive rates between Wuhan and Chongqing could be explained by the incidence rates in the two cities, as Wuhan was the epicenter of the epidemic. Moreover, the definition of close contacts in the Protocol on Prevention and Control of Novel Coronavirus Pneumonia (Edition 6) included close contacts of patients with suspected cases, which significantly decreased the NAT positive rate. The positive rate of close contacts was 8.00% in Chongqing, which was comparable to 6.15% in Ningbo (1). Neither Ningbo nor Chongqing were epidemic outbreak centers. In addition, the positive rate of NAT was 0% for 1,612 people in special institutes, such as prisons, pension agencies, and other institutions; therefore, NAT of samples from people associated with these institutes may be not as urgent as testing of samples from people in high-risk exposure groups. However, 81.82% (18/22) of cases were positive on the first nucleic acid test, and only four of 22 cases were diagnosed after two or three tests, which showed that NAT has good sensitivity for detecting SARS-CoV-2. Our results suggest that different detection ranges for NAT should be defined in the diagnostic guidelines according to the level of severity of the COVID-19 epidemic in each location to improve the positive detection rate and conserve and rationally allocate medical resources.

Variants of SARS-CoV-2 have imposed new challenges in disease control and further prove the importance of nucleic acid detection. The delta variant of SARS-CoV-2 has caused resurgence in COVID-19 epidemics in many countries. Accelerating the popularization of vaccination, improving the coverage rate, and implementing intervention measures are effective means to control the spread of SARS-CoV-2 variants. However, vaccination against SARS-CoV-2 alone without intervention measures may lead to continuous spread and the emergence of new variants (39). The delta and lambda variants exhibit changes in nonstructural proteins (NSPs) and the S protein compared to the original Wuhan strain. The lambda variant also has numerous amino acid substitutions in NSPs and S proteins, plus a deletion in the N-terminal domain (NTD) of the S protein, leading to partial escape from neutralizing antibodies (NAbs) in vaccinated individuals. The S protein is one of the most mutable parts of the SARS-CoV-2 genome. The investigation of alternative protein targets other than spike-based protein targets or treatments to stimulate an immune response is suggested (40). Three receptor-binding domain (RBD)-specific monoclonal antibodies (mAbs), 58G6, 510A5, and 13G9, with high neutralizing potency against authentic SARS-CoV-2, have remarkable efficacy against authentic B.1.351 virus (41). During this pandemic, human behavior has strongly affected the adaptive process of SARS-CoV-2 through continuous iterations and changes to implemented control measures. Accurate detection is required for SARS-CoV-2 infection diagnosis throughout the whole epidemic period. Many nucleic acid tests based on RT-PCR have been developed, each with different techniques, specifications, and turnaround times. As local epidemics progressed to a pandemic, testing is more crucial. For surveillance, serologic testing was necessary (42), and the IgM-IgG antibody test was a useful adjunct to RT-PCR detection and improved the accuracy of COVID-19 diagnosis regardless of the severity of illness. The application of serological testing to assist in confirming SARS-CoV-2 infection detected by viral NAT is recommended, especially when COVID-19-related symptoms are present and viral nucleic acid test results are negative (9, 12, 43). The increase in COVID-19-associated waste (CAW) and its presence in the environment will result in easy access by other organisms, and there is a great demand for an efficient strategy to prevent further spread in the environment (44, 45).

The epidemiological characteristics of the 22 cases provide data for the prevention and control of COVID-19 in other countries worldwide affected by the pandemic. The incubation period of the 21 symptomatic cases was comparable to that in the study by Li Q et al. (46), the median recovery duration was 22 days, and the longest recovery duration was 37 days. There were more female patients than male patients in this study, which was inconsistent with the results from other studies (47). Five cases were imported from Wuhan, or patients had a travel history to Wuhan. The proportion of local secondary cases (77.27%) was higher than that in Gansu Province (48), as the population density in Gansu is less than that in Chongqing, suggesting that the transmission of secondary cases was serious in high population-density districts. In addition, 95.45% of the cases were part of a cluster, which indicate human-to-human transmission (49, 50). Moreover, one decedent who worked in catering transmitted COVID-19 to 13 people; this case was not diagnosed until after death, indicating that the decedent was highly infectious, making it difficult to interrupt the chain of transmission. People working in restaurants and supermarkets are part of the high-risk population (51), and quarantine should be carried out if there are suspected cases in this population. Moreover, NAT should be carried out for suspected cases, even if the patient has died. A SARS-CoV-2 nucleic acid test was positive after 28 days (including 14 days of centralized isolation) in one patient who had recovered, and the positive result was found when donating blood after recovery. In addition, an anal swab from an asymptomatic carrier was positive at 41 days after diagnosis, which was consistent with the results in children (52). The asymptomatic carrier was younger (18 years old) than the symptomatic patients (53). Therefore, it is necessary to be aware of the possibility of fecal-oral transmission of COVID-19, and increasing surveillance among asymptomatic carriers and recovered patients after discharge from the hospital will reduce community transmission of COVID-19.

SARS-CoV-2 may be transmitted through close contact with an infected person (54), droplets, and aerosols (55). Therefore, identifying close contacts of patients through epidemiological surveys is important in controlling the COVID-19 epidemic (56). Our results showed that close contacts were mainly family members, relatives, friends, and coworkers (53), whereas some were strangers who had contact with the patient by accident; the latter are difficult to track. Public security agencies have provided a substantial amount of data to track accidental close contacts.

Public health interventions for COVID-19 have some limitations. First, investigating the close contacts of people with suspected cases may increase the burden on the CDC, as suspected cases should be diagnosed within 3 days if they have been infected with SARS-CoV-2. Second, the positive rate of NAT was very low, as many people who had no contact history or clinical symptoms received NAT. The clinical guidelines for COVID-19 should consider the severity level of the COVID-19 epidemic.

In conclusion, effective public health interventions were implemented to constrain the spread of COVID-19 in China. The positive rate of SARS-CoV-2 NAT was very low for the total population, but it was higher in those with suspected cases and close contacts. Therefore, more effective detection ranges should be defined to increase the positive detection rate. Those who recover from COVID-19 may become positive asymptomatic carriers, as SARS-CoV-2 NAT was positive for an asymptomatic carrier at 41 days after diagnosis. One patient was diagnosed after death. Therefore, increasing surveillance of SARS-CoV-2 *via* NAT of asymptomatic carriers recovered individuals after discharge from the hospital and patients with suspected cases who die will reduce community transmission. Moreover, this study provides policy suggestions for how to quickly detect positive cases of acute respiratory infectious diseases at the beginning of an outbreak, what types of populations should be screened first, and how to effectively prevent missed diagnoses and reduce transmission by those who died from COVID-19.

## Data Availability Statement

Publicly available datasets were analyzed in this study. This data can be found at: www.nhc.gov.cn.

## Ethics Statement

The studies involving human participants were reviewed and approved by Children's Hospital of Chongqing Medical University. Written informed consent for participation was not required for this study in accordance with the national legislation and the institutional requirements.

## Author Contributions

XL conceived and designed the experiments and wrote the paper. LX, YS, YR, and XT performed the experiments and participated in the epidemiological survey. All authors revised the manuscript, critically reviewed and approved the final paper.

## Funding

This work was supported by the Technology Foresight and Institutional Innovation Project of Chongqing Science and Technology Bureau (number cstc2020jsyj-zzysbAX0016), Joint Medical Research Project of Chongqing Municipal Health Commission and Chongqing Science and Technology Bureau (2020FYYX142); Chongqing Medical University Funded Projects (CQMUNCP0204), the National Key Research and Development Project [2017YFC0211705], the Natural Science Foundation of Youth Project [81502826], the Education Commission of Chongqing Municipality (KJQN201900443), the Science and Technology Bureau of Jiulongpo District Foundation [2019-02-001-Z], the China Postdoctoral Science Foundation [2014M562289], and Chongqing Postdoctoral Research Funded Projects [Xm2014129].

## Conflict of Interest

The authors declare that the research was conducted in the absence of any commercial or financial relationships that could be construed as a potential conflict of interest.

## Publisher's Note

All claims expressed in this article are solely those of the authors and do not necessarily represent those of their affiliated organizations, or those of the publisher, the editors and the reviewers. Any product that may be evaluated in this article, or claim that may be made by its manufacturer, is not guaranteed or endorsed by the publisher.
